# Depressive Symptoms and Cortisol Rhythmicity Predict Survival in Patients with Renal Cell Carcinoma: Role of Inflammatory Signaling

**DOI:** 10.1371/journal.pone.0042324

**Published:** 2012-08-01

**Authors:** Lorenzo Cohen, Steven W. Cole, Anil K. Sood, Sarah Prinsloo, Clemens Kirschbaum, Jesusa M. G. Arevalo, Nicholas B. Jennings, Shellie Scott, Luis Vence, Qi Wei, Diane Kentor, Laszlo Radvanyi, Nizar Tannir, Eric Jonasch, Pheroze Tamboli, Louis Pisters

**Affiliations:** 1 Department of General Oncology and the Integrative Medicine Program, The University of Texas MD Anderson Cancer Center, Houston, Texas, United States of America; 2 Department of Medicine, Division of Hematology-Oncology, University of California Los Angeles School of Medicine, Los Angeles, California, United States of America; 3 Department of Gynecologic Oncology and Cancer Biology Center for RNA Interference and Non-Coding RNA, The University of Texas MD Anderson Cancer Center, Houston, Texas, United States of America; 4 Technical University of Dresden, Dresden, Germany; 5 Department of Urology, The University of Texas MD Anderson Cancer Center, Houston, Texas, United States of America; 6 Department of Melanoma Medical Oncology, The University of Texas MD Anderson Cancer Center, Houston, Texas, United States of America; 7 Department of Genitourinary Medical Oncology, The University of Texas MD Anderson Cancer Center, Houston, Texas, United States of America; 8 Department of Pathology, The University of Texas MD Anderson Cancer Center, Houston, Texas, United States of America; University of Pennsylvania, United States of America

## Abstract

**Purpose:**

Evidence has supported the association between psychological factors and cancer biology; however, findings are equivocal on the role of psychosocial factors in cancer progression. This study generates a hypothesis of mechanistic variables by examining the clinical effects of psychosocial factors and cortisol dysregulation in patients with metastatic renal cell carcinoma (RCC) and examines associated activation of transcription control pathways.

**Methods:**

Patients with metastatic RCC (n = 217) were prospectively enrolled in this study. Patients completed questionnaires (Centers for Epidemiologic Studies – Depression; SF-36 Health Status Survey; Duke Social Support Index; Coping Operations Preference Enquiry; organized and non-organized religious activity; and intrinsic religiosity), and provided blood and saliva samples. Cortisol levels and whole genome transcriptional profiling were assessed to identify potential alterations in circadian rhythms and genomic pathways.

**Results:**

Separate Cox regression models, controlling for disease risk category, revealed that CES-D scores (p = 0.05, HR = 1.5, 95% CI for HR: 1.00–2.23) and cortisol slope (p = 0.002; HR = 1.9; 95%CI for HR: 1.27–2.97) were significantly associated with decreased survival. Only cortisol slope and risk category remained significant in the complete model. Functional genomic analyses linked depressive symptoms to increased expression of pro-inflammatory and pro-metastatic genes in circulating leukocytes. 116 transcripts were found to be upregulated by an average of 50% or more in high CES-D patients, and 57 transcripts downregulated by at least 50%. These changes were also found in the tumor in a subset of patients.

**Conclusion:**

These findings identify depressive symptoms as a key predictor of survival in renal cell carcinoma patients with potential links to dysregulation of cortisol and inflammatory biology.

## Introduction

Evidence shows that psychosocial factors may contribute to cancer progression via multi-factorial biological pathways; however mechanisms of psychological stress in cancer survival remain controversial and poorly understood, especially in clinical contexts [1], [2], [3], [4], [5]. Recent research has found that stress and depression may exert biological effects by increasing pro-inflammatory signaling [6] and a separate literature has linked inflammatory gene expression within the tumor microenvironment to increased angiogenesis, invasion, and metastasis [2]. Although there is research showing that depression is associated with worse survival in cancer [7], controversy still exists around the interpretation that psychosocial and biological factors can indeed contribute interdependently to disease processes [8]. However, some research exists that shows that biological systems are influenced by previous or ongoing psychological symptoms in animals and humans [3]. Nevertheless, in human studies it remains difficult to determine causality. Therefore, we designed this prospective study to further explore the interplay between patient psychological condition and the progression of advanced stage renal cell carcinoma, and to define the role of inflammatory gene expression and its regulation by glucocorticoid hormones as potential mediators of such effects. We sought to answer previously unanswered questions about the potentially reciprocal relationships between multiple psychological and other biological variables and their independent and interrelated association to cancer progression.

Associations between depression and other psychological variables with alterations in hormone function could potentially affect inflammation and cancer progression [9], [10], [11], [12], [13], [14]. The central nervous system’s control of glucocorticoids (including cortisol) provides one link between the nervous and endocrine systems. For example, individuals with major depressive disorder have been found to have increased levels of cortisol, one indication of dysregulation in the endocrine system [13]. Cortisol binds to the glucocorticoid receptor in the cytoplasm and is then translocated into the nucleus where it modulates gene transcription, leading to cellular changes. Both depression and cortisol have been linked as mechanisms that may lead to a hypoactivity of the glucocorticoid receptors on immune cells and in limbic areas of the brain [15]. Glucocorticoids may enhance circulating levels of cortisol and therefore depression is thought to result in hypersecretion of pro-inflammatory cytokines and increased activity of the HPA axis [6]. Additionally, there is some evidence that alternations in cortisol levels and cortisol rhythms are associated with tumor progression [16] and decreased survival [17]. Several previous studies have also linked depression and other types of experienced adversity to increased leukocyte expression of pro-inflammatory cytokines and other gene products that can potentially contribute to cancer progression via the expression of pro-metastatic genes by tumor-associated macrophages [18], [19], [20]. These effects are associated with increased activity of pro-inflammatory NF-κB (nuclear factor kappa-light-chain-enhancer of activated B cells) and STAT (signal transduction and transcription) family transcription factors [21], as well as EGR(early growth response) and MEF(myocyte enhance factor) and MZF (myeloid zinc finger) family transcription factors involved in myeloid cell differentiation and activation [22], [23]. The hypotheses in the present study were that: 1) psychological symptoms (depressive symptoms, social support, coping, and religiosity/spirituality) at time of diagnosis with metastatic RCC will be associated with survival time; 2) systemic cortisol dysregulation will be associated with survival time; and 3) systemic cortisol dysregulation and inflammatory gene expression will contribute to the association between psychological symptoms, survival, and tumor-based parameters.

## Methods

Participants were 217 patients with newly diagnosed metastatic RCC at the University of Texas MD Anderson Cancer Center, a life expectancy of greater than 4 months, a Zubrod performance status of less than or equal to 2, and no serious intercurrent illnesses. Informed consent was obtained from each participant prior to enrollment in the study. Period of protocol accrual was from April 2000 through November 2005. At the time of enrollment patients completed a battery of questionnaires, provided a blood sample, and collected five saliva samples per day for the subsequent three consecutive days (upon awakening, 45 minutes later, 8 and 12 hours after waking, and at bedtime). The study was approved by the Surveillance Committee for the Protection of Human Subjects at MD Anderson. Appropriate material transfer agreements were in place for the assessment of the blood and saliva samples.

At study entry, patients completed several psychosocial questionnaires. The Centers for Epidemiologic Studies - Depression was used to assess depressive symptoms, with scores of 16 or above classified as meeting screening criteria for depressive symptoms with further evaluation recommended [24]. We chose to dichotomize the CES-D based on the well-established cut-off scores for screening criteria to avoid artificially creating cut off scores for a continuous measure, as well as for ease of reporting and interpreting results. Moreover, our interest was in determining whether patients who meet a minimum threshold for depressive symptoms would have worse outcomes and not examining symptom severity on a continuum, which would have less clinical relevance. Furthermore, a number of past meta-analyses examining the role of depressive symptoms on biological and economic outcomes have excluded studies that reported depressive symptoms as a continuous outcome [25], [26]. The SF-36 Health Status Survey assessed quality of life [27] (Physical Component Scores and Mental Health Component Scores are reported). The Duke Social Support Index [28] contains two subscales and measures size and structure of social network, and perceived satisfaction with support obtained from the network [29] and the Coping Operations Preference Enquiry (Brief-COPE) [30] which measures preferences for certain coping mechanisms, were also completed. For the B-COPE, we calculated subscales for Engagement and Avoidant coping [31]. We also used a multi-modal assessment of religiosity/spirituality including measuring organized religious activity, non-organized religious activity [32], and intrinsic religiosity [33]. Medical information was abstracted from medical charts.

Saliva samples were frozen and then shipped to Dr. Clemens Kirschbaum, Department of Psychology, Dresden University of Technology, for cortisol assay. Levels of cortisol were determined using a time-resolved immunoassay with fluorescence detection.

Blood samples were collected in sterile heparinized tubes (30 ml total) between 7–11 am and PBMCs (peripheral blood mononuclear cells) were isolated by Ficoll-Hypaque gradient centrifugation and cryopreserved. Gene expression profiling was carried out using total RNA extracted (Qiagen RNeasy) from PBMCs from a subgroup of 31 patients (15 patients with highest CES-D scores (≥16) and 16 patients with the lowest CES-D scores (<16) – matched on: sex, age at metastatic disease, smoking history, and disease risk group - low, intermediate, and high based on the following risk factors: Karnofsky <80%; corrected calcium > = 10; serum hemoglobin < = 13 mg/dl for males and < = 11. 5 mg/dl for females; serum lactate dehydrogenase (1.5 times the upper limit of normal –upper limit of normal was 618 IU/L), previous radiation therapy; number of metastatic sites > = 2; and interval between date of diagnosis and date of registration < = 1 year [34]. Those with 0 or 1 risk factor were classified at low risk, those with 2 risk factors were classified at intermediate risk, and those with more than 2 risk factors were classified at high risk) [34].

All samples met quality assurance standards for RNA mass and integrity, and whole genome transcriptional profiles were assayed by Illumina Human Ref-8 BeadArrays in the UCLA Social Genomics Core and the UCLA Southern California Genotyping Consortium, following the manufacturer’s specified protocol (Illumina Inc., San Diego CA). Data were quantile normalized and log-2 transformed for differential expression analyses identifying transcripts showing ≥50% difference in average expression across groups while controlling False Discovery Rates at 5%. Data are posted as Gene Expression Omnibus series GSE36957. Differential gene expression was determined using a fold-change cut-off because previous studies have shown that fold-change thresholds yield more replicable results than do p-value-based thresholds [35], [36], [37], [38], [39], [40]. To avoid potential confounding with correlates of CES-D scores, all differential gene expression analyses controlled for patient age, sex, ethnicity, education, marital status, body mass index, and disease risk index. Functional commonalities among differentially expressed genes were analyzed by GOstat Gene Ontology analysis (with default parameter settings) [41], and promoter-based bioinformatics analyses were carried out to identify transcription control pathways mediating the observed effects [42] (both controlling False Discovery Rates at ≤5%). Promoter-based bioinformatics focused on the hypothesis that high CES-D scores would be associated with increased expression of genes with promoters bearing predicted binding sites for the pro-inflammatory NF-κB and STAT transcription factors (assessed by Transfac V$NFKB_Q6 and V$STAT1_01 nucleotide weight matrices, respectively) and transcription factors involved in monocyte/macrophage activation (V$EGR1_01-V$EGR3_01, V$NGFIC_01, V$MEF2_02, and V$MZF1_01) [22]. Results represent the mean fold-difference in promoter response elements for each of those 8 transcription factor-binding motifs averaged over 9 different combinations of 3 promoter lengths (−300, −600, and −1000 to +200 bp relative to gene transcription start site) and 3 transcription factor motif detection stringencies (Transfac mat_sim  = .80, .90, .95) [42].

**Table 1:** Demographic and medical characteristics and Cox semi-parametric regression models for survival from diagnoses with metastatic disease (N = 202).

Variable	Mean/Percent (SD)	Hazard Ratio	95% CI	P value
Age	59 (10)			
> = 60	49%	1.03	(0.7, 1.5)	0.87
<60	51%			
Sex				
Male	77%	1.09	(0.7, 1.7)	0.68
Female	23%			
Karnofsky score <80%	9%	2.66	(1.6, 4.5)	0.0003
Corrected calcium> = 10	5%	1.76	(0.9, 3.5)	0.10
Low HBG	40%	1.79	(1.3, 2.5)	0.001
Previous radiation therapy	10%	1.14	(0.7, 2.0)	0.65
Metastatic sites > = 2	64%	1.37	(0.9, 2.0)	0.10
Time from diagnosis < = 1 year	60%	1.82	(1.3, 2.6)	0.002
Pathology				
Sarcomatoid	8%	3.13	(1.8, 5.5)	0.0005
Papillary	8%	1.13	(0.6, 2.2)	
Other	11%	0.65	(0.3, 1.4)	
Clear cell	74%			

SD  =  Standard Deviation.

Formalin-fixed, paraffin-embedded tissues were used for immunohistochemical-peroxidase staining for macrophages (CD68), HIF1α, MMP-2, MMP-9, or COX-2, as previously described [19], [43].

Statistical Analyses: The database was locked for analyses May 2010. Correlation coefficients between study variables were first determined. For the purposes of this paper, we examined baseline psychosocial factors and cortisol slope as predictors of survival. Cox semi-parametric regression models were utilized to model survival from the diagnoses with metastatic disease of the participants and examine the univariate association of psychosocial factors, cortisol slope, demographic, and medical factors. As mortality is commonly associated with the metastasis of disease [44] rather than the primary tumor, we chose to conduct our analysis from the time of diagnosis of metastatic disease versus initial diagnosis with earlier stage of disease. In order to have the cortisol data normally distributed, cortisol raw score levels were log-transformed. Cortisol slopes were calculated by regressing log-transformed cortisol levels on saliva collection time (five times a day for three days) for each patient. For all subsequent analyses, we included RCC risk factor classified as low, intermediate, or high risk as described above. We then analyzed separately models including CES-D, SF-36 PCS, and cortisol slopes, as each were associated with survival in the univariate analyses. We also ran Cox regression models to determine the associations between cortisol and depressive symptoms on survival in the same model in order to examine cortisol slope as a potential mediator. Model assumptions were evaluated for all variables using standard residual-based diagnostic procedures.

## Results

Demographic, medical, and psychosocial variables were available for 202 patients (Table 1). At the time of analysis, 64% of patients were deceased. For those who had died, the average time from diagnosis of metastatic disease to death was 1.8 years (SD = 1.31, range 0.25–6.25 years). Mean CES-D scores were 10.2 (8.1), with 23% of the population scoring 16 or above. Of the 202 patients with complete medical and psychosocial data, 129 provided complete saliva data for cortisol analyses. We examined for possible differences in the patients who did and did not provide saliva samples in regard to demographic and medical characteristics and CES-D scores. The only differences between the patients who did and did not provide saliva samples is that the patients who did not provide saliva samples were on average younger (56 vs 61 years; p = .001) and would thus be expected to show greater average survival [45]. There were no differences in survival between the patients who did and did not provide saliva samples.

**Figure 1:**
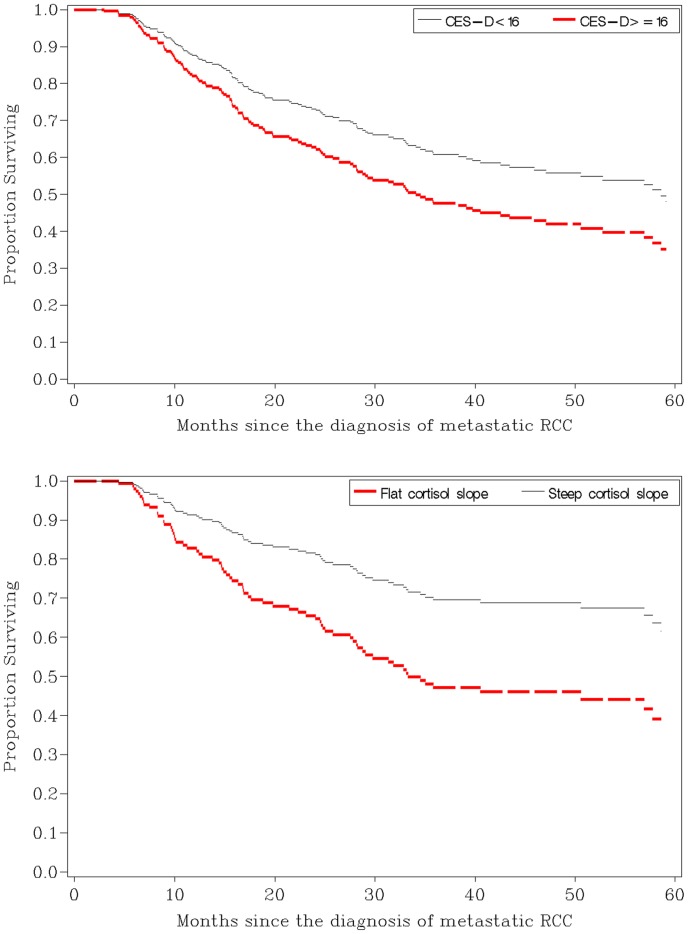
This figure shows proportional survival curves adjusted for risk category. CES-D scores were categorized as greater or equal to 16 or less than 16 (p = 0.05, HR = 1.5, 95% CI for HR: 1.0–2.2). Survival function of 1 SD above and below the raw means was estimated for cortisol slope (p = 0.002; HR = 1.9; 95%CI for HR: 1.3–3.0).

As shown in Table 2, a series of univariate survival analyses were conducted to examine the association between individual variables and survival time from the diagnoses of metastatic disease. The following factors were associated with survival time: CES-D scores, SF-36 PCS scores, cortisol slope, and risk category. None of the other psychosocial variables were associated with survival (see Table 2). Separate Cox regression models, controlling for risk category, showed that CES-D scores and cortisol slope were still associated with survival (see Figure 1) and SF-36 PCS scores was no longer associated with survival. To determine whether cortisol dysregulation might mediate the effects of depressive symptoms as a risk factor for mortality, we then examined the association of CES-D scores and cortisol slope in the same model (Table 3). Results showed that the association between CES-D scores on survival was no longer significant when cortisol slope was in the model, and cortisol slope and risk category remained associated with survival (Table 3). All results remained the same when one patient who was still alive 12 years after diagnosis of metastatic disease was excluded from the analyses or if patients were excluded if they had died within 6 months of study entry. CES-D scores remained a significant predictor of survival in models controlling for risk category and SF-36 PCS scores (a measure of performance status) or risk category and the treatment patients received (chemotherapy, radiotherapy, immune therapy, or biological therapy) (data not shown). Correlation analyses revealed there was no association between the psychosocial variables and cortisol slope. Importantly, there was also no association between CES-D scores or cortisol slope and treatment-related factors or risk category (data not shown).

**Table 2:** Cox semi-parametric regression models for survival from diagnosis with metastatic disease (N = 202).

Variable	Mean/Percent (SD)	HazardRatio	95% CI	Pvalue
CES-D	10.2 (8.1)			
> = 16	23%	1.75	(1.2, 2.6)	0.005
<16	77%			
PCS	37.4 (11.9)	0.98	(0.9, 1.0)	0.01
MCS	52.1 (9.9)	0.99	(0.9, 1.0)	0.42
DSSI	39.3 (17.8)	1.01	(0.9, 1.0)	0.06
Engagement coping	24.2 (5.1)	1.01	(0.9, 1.1)	0.39
Avoidant coping	5.5 (2.5)	1.04	(0.9, 1.1)	0.25
ORA	9.4 (4.5)	0.99	(0.9, 1.0)	0.48
NORA	11.4 (5.6)	0.99	(0.9, 1.0)	0.62
HOGE	2.2 (0.9)	1.1	(0.9, 1.3)	0.38
Cortisol slope	−0.7 (0.6)	1.88	(1.3, 2.8)	0.002
Risk group				<0.0001
Poor risk	28%	2.90	(1.9, 4.4)	
Intermediate risk	29%	1.68	(1.1, 2.6)	
Favorable risk	44%			

SD  =  standard deviation; CES-D  =  Centers for Epidemiologic Studies-Depression; PCS  =  SF-36 Health Status Survey Physical Component Scores; MCS  =  SF-36 Health Status Survey Mental Health Component Scores. DSSI  =  Duke Social Support 11 items total score; ORA  =  Organized religiousness; NORA  =  Private religious practices; HOGE  =  Intrinsic religious motivation scale.

**Table 3:** Multivariate Cox regression models for survival from the diagnosis with metastatic disease.

Variable	ParameterEstimate (SE)	HazardRatio	95% CI	P value
CES-D	0.27 (0.31)	1.32	(0.7, 2.4)	0.37
Risk group				0.02
Poor risk	0.83 (0.29)	2.29	(1.3, 4.1)	
Intermediate risk	0.42 (0.28)	1.52	(0.9, 2.7)	
Favorable risk				
Cortisol slope	0.67 (0.22)	1.96	(1.3, 3.0)	0.002

SE  =  standard error; CES-D  =  Centers for Epidemiologic Studies - Depression.

**Figure 2:**
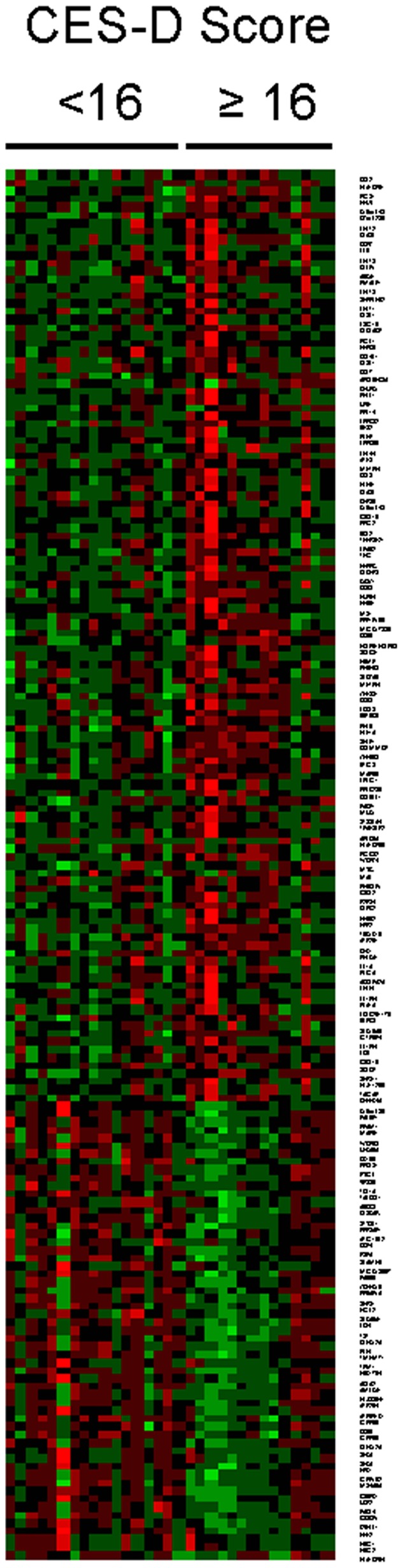
This figure shows differential leukocyte gene expressions in patients scoring greater or equal to 16 versus less than 16 on the CES-D.

To determine whether the increased mortality risk associated with elevated CES-D scores might stem from increases in pro-inflammatory gene expression in the immune system, whole-genome transcriptional profiling was carried out on circulating PBMC samples from 15 patients with the highest levels of depressive symptoms (CES-D ≥16; mean (SD) = 25. 1(6.9); range  = 16–35) and 16 demographically and disease risk-matched patients with the lowest CES-D scores (CES-D <16; mean (SD) = 3.2 (2.9); range  = 0–8) (Figure 2). 116 transcripts were found to be upregulated by an average of 50% or more in high CES-D patients, including transcripts encoding pro-inflammatory mediators and their indicators (COX2/*PTGS2*, *IL6*, *TNF*, *IL1A*, *IL1B*, *IL1RN*), indicators of oxidative stress (*SOD2*), chemokines and their receptors (*CCL2*, *CCL3*, *CCL3L1*, *CCL4L1*, *CCL7*, *CCL8*, *CCL20*, *CCR7*, *CXCL1*, *CXCL16*), Type I interferons and associated response genes (*IFNB1*, *IFI44*, *IFIT1*, *IFIT2*, *IFIT3*, *ISG15*, *OASL*), indicators of leukocyte activation (*CD69*, *HLA-DR*, *CD83*, *BCL2*), and the hypoxia response gene *HIF1A*. The 57 transcripts downregulated by at least 50% included leukocyte differentiation antigens (*CD24*, *CD36*, *CD79B*) and the fractalkine receptor *CX3CR1*. Gene Ontology analyses identified upregulation of genes involved in inflammation, immune response, and negative regulation of programmed cell death (all p<.0001) and downregulation of genes involved in cell trafficking, adhesion, oxygen transport, and hemostasis (all p<.05). Promoter-based bioinformatics analyses indicated increased activity of the pro-inflammatory NF-κB (1.75±.07-fold upregulation; p = .002) and STAT1 transcription factors (1.80±.13-fold upregulation; p = .045), as well as increased activity of multiple factors involved in myeloid cell differentiation and activation (EGR1∶2.44±.11-fold; p = .013; EGR2∶4.09±.09-fold; p = .003; EGR3∶3.34±.16-fold; p = .015; EGR4/NGFIC: 1.99±.08-fold, p = .0125; MEF2∶1.84±.06-fold; p = .009; MZF1∶1.51±.08-fold; p = .004).

**Figure 3:**
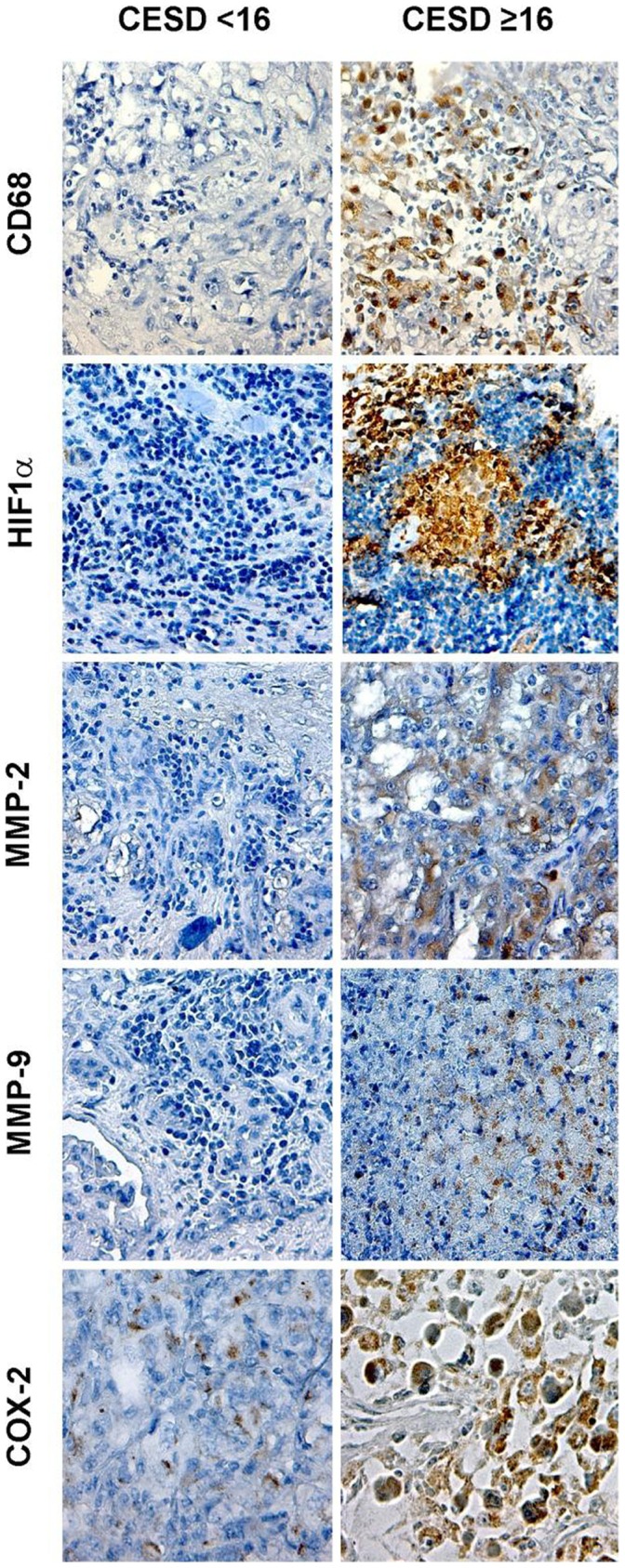
This figure shows representative images of immunohistochemical staining for CD68, HIF1α, MMP-2, MMP-9, and COX-2 in patients scoring greater or equal to 16 versus less than 16 on the CES-D. Pictures were taken at original magnification X200.

To determine whether changes associated with inflammation were present in the tumor microenvironment, we examined 10 patient’s tumor tissue samples for tumor-associated macrophages (TAMs; CD68) and other protein markers using immunochemistry (from the matched patients for whom we did gene array analyses; (N = 3 CES-D ≥16; N = 7 CES-D <16). Individuals with high CES-D scores had significantly greater TAMs compared to those with low scores (mean 61.9 *versus* 25.3). There was also higher expression of HIF1α, MMP-2, MMP-9, and COX-2 in tumors for patients with high CES-D scores (Figure 3).

## Discussion

Our findings provide the first evidence that levels of depressive symptoms are associated with survival time among patients with newly diagnosed advanced RCC, controlling for disease-and treatment-related factors. The present analyses also found an association between a blunting of normal diurnal variation in cortisol levels to increased risk of mortality. Statistical control for flattened diurnal cortisol rhythm substantially reduced the association between depressive symptoms and survival, indicating that cortisol dysregulation may potentially play a role in mediating the association between depressive symptoms and survival. In fact, cortisol slope was as predictive as disease risk categorization. Although previous research has shown that depression and cortisol slope are associated with survival in cancer patients [17], this study provides new evidence that those two dynamics might be related, and that altered cortisol regulation might partially mediate the relationship between depressive symptoms and cancer progression.

Gene expression profiling of circulating immune cells from patients with elevated depressive symptoms was consistent with this hypothesis in identifying marked upregulation of pro-inflammatory genes (e.g., *IL1B, TNF, IL6, PTGS2*) and associated transcriptional regulators (e.g., NF-κB and STAT family transcription factors) that have previously been linked to cancer progression and metastasis via tumor-associated macrophages [46]. For example, IL-6 is a growth and survival factor for many tumor types [47]. In renal cell carcinoma, increased IL-6 production has been shown to correlate with expression of tumor suppressor gene proteins [47], and may be involved in the persistent activation of STAT transcription factors [23]. Likewise, hypersecretion of IL-6 and other proinflammatory cytokines has been shown in the pathophysiology of psychologically based symptoms [6], [48], [49]. Histological analyses of primary tumor tissues from a random subset of patients confirmed differences in macrophage infiltration and pro-inflammatory mediators at the protein-level. Given the key role of the HPA axis and cortisol in regulating inflammatory biology, the functional dysregulation of cortisol production observed here could allow the development of pro-metastatic gene expression profiles in the circulating monocyte pool that ultimately interacts with the primary tumor to promote metastasis [50]. Altered HPA axis activity may also facilitate RCC disease progression by dysregulating anti-tumor cellular immune responses [1].

There are a number of limitations with the current study that are important to note. Although there have been numerous animal studies indicating a link between psychological stress and cancer progression, it is a challenge to determine causality in human models, as it is unethical to induce stress. While our gene expression data provides support for a more causal role of depression predicting survival, our current study design cannot assess causality. For example, aspects of the tumor microenvironment associated with peripheral markers of inflammation and prognosis could be influencing depressive symptoms versus the association being in the other direction. It is also possible that there are other factors related to disease severity or other indicators of health that could conceivably influence both levels of depressive symptoms and survival that were not assessed in the current study. However, we controlled for disease risk factors, treatment, and physical function. In addition, our sample was homogenous in terms of disease status (all stage IV disease, low calcium levels, and low Zubrod scores). A further limitation is that the measures of depressive symptoms were assessed following the patients’ diagnosis with cancer. Whether levels of distress or depressive symptoms were present prior to or following the patients’ cancer diagnosis could not be determined. However, depressive symptoms were not associated with disease-related risk factors or the treatment patients underwent. However, some previous research has shown that cancer patients who have emotional problems prior to their diagnosis have shorter survival than those with an onset of symptoms following diagnosis [51]. The timing of the onset of depressive symptoms could not be assessed in the current study, and this is an area that warrants future investigation. Another limitation of our study is the small sample size for the genome transcriptional profiling. However, our findings are consistent with preclinical models regarding the effects of biopsychosocial stress on tumor biology [1]. In light of the limitations of this study, our results should be interpreted with caution and confirmed through replication in future trials.

Collectively, the present data suggest that the association between RCC patient psychological condition and survival time may stem from systemic dysregulation of inflammatory biology resulting from blunted cortisol rhythmicity and subsequent de-repression of pro-inflammatory signaling pathways within the tumor microenvironment that subsequently contribute to disease progression and metastasis. While depressive symptoms, inflammatory markers, immune function, and gene regulation have been shown to be independently linked to the progression of cancer and survival, the current study is the first to demonstrate a more systemic model of cancer that includes both central and peripheral nervous system function and the influence of psychological well- being on the system. Defining the neural and hormonal mechanisms of such effects could provide new biological targets for adjunctive control of disease progression in the context of RCC.
